# Demographic and modifiable risk factors impacting obstructive sleep apnea comorbidities: a New Orleans case–control study

**DOI:** 10.1007/s11325-025-03379-z

**Published:** 2025-06-06

**Authors:** Ajibola B. Bakare, Diandra Smith, Zaynab Sidi Mohamed, Tijani Mohammed, Mina Liao, Brian Le, Rizwan Aslam

**Affiliations:** 1 Tulane University School of Medicine, 1430 Tulane Ave, New Orleans, LA 70112 USA; 2 Department of Otolaryngology Head and Neck Surgery, Tufts Medical Center, Boston, MA 02111 USA; 3 Department of Otolaryngology, Tulane University School of Medicine, New Orleans, LA 70112 USA

**Keywords:** Cardiovascular disorders, Comorbidities, Demographics, Metabolic disorders, Modifiable Risk Factors, Obstructive sleep apnea

## Abstract

**Purpose:**

Obstructive sleep apnea (OSA) is a prevalent sleep disorder characterized by recurrent upper airway collapse during sleep, leading to intermittent cessation or reduction of breathing. Associated symptoms include snoring and excessive daytime sleepiness. OSA is linked to a range of comorbidities, primarily cardiovascular and metabolic conditions. However, emerging evidence suggests a broader spectrum of associated health issues. This study aimed to investigate the relationship between OSA comorbidities and demographics as well as modifiable risk factors within a New Orleans patient population.

**Methods:**

A case–control study encompassing 19,875 patients (313 with OSA, 19,558 without) was conducted. Nominal logistic regression analyzed differences between OSA and non-OSA groups, while prevalence of comorbidities was compared across demographic and modifiable risk factors within each group.

**Results:**

Our findings revealed increased odds of cardiovascular and respiratory disorders among OSA patients compared to controls. OSA risk was elevated in males, middle-aged individuals, individuals with body mass index (BMI) greater than 30, and former smokers. Cardiovascular and metabolic conditions were the most prevalent comorbidities in the OSA group, with additional comorbidities including gastrointestinal, respiratory, psychiatric, and renal disorders.

**Conclusion:**

These results highlight the multifaceted health burden associated with OSA. Comprehensive management strategies addressing both well-established and emerging comorbidities are essential for optimizing patient outcomes.

**Supplementary Information:**

The online version contains supplementary material available at 10.1007/s11325-025-03379-z.

## Introduction

Obstructive sleep apnea (OSA) is a common sleep disorder characterized by recurrent upper airway collapse during sleep, causing apneas or hypopneas. These interruptions often accompanied by snoring and excessive daytime sleepiness, lead to sleep fragmentation and oxygen deprivation, impacting overall health [1–3]. In the United States, OSA affects 48% of the population, with nearly 80% of cases undiagnosed [4]. Its rising prevalence highlights a significant public health burden [5].

Recent studies have highlighted the complex interplay between OSA and comorbidities beyond cardiovascular and metabolic manifestations [1, 3, 6]. Sheu et al. identified a link between OSA and sudden sensorineural hearing loss in men [6], while other studies have revealed a broader spectrum of associated conditions, including respiratory, and metabolic disorders, which contribute to increased mortality risk in OSA patients [7]. OSA is linked to a growing list of comorbidities including metabolic (diabetes and obesity), respiratory (asthma and chronic obstructive pulmonary disease (COPD)), neurological (stroke, cognitive impairment, neurodegeneration, epileptic seizures), gastrointestinal (GI), and mental health disorders [1, 3, 6, 8–10]. The bidirectional nature of this relationship, where OSA influences comorbidities and vice versa, underscores its complexity. Each of these comorbidities is associated with increased mortality in OSA patients compared to age-matched individuals in the general population [7, 11]. OSA is more prevalent in males and associated with increasing age [2, 9, 12–14]. Modifiable risks factors such as obesity, tobacco and alcohol use have also been associated with OSA [1, 15–18]. Although obesity is a well-established risk factor for OSA and continues to rise in the U.S., its impact appears to decline after age 60 [19]. Similarly, gender differences in OSA are reported to diminish after menopause, with rates in postmenopausal women approaching those seen in men [20]. Interestingly, despite lower average BMIs in Asian populations relative to Western populations, OSA prevalence remains similar, suggesting that non-obesity-related factors also contribute to its pathogenesis [20]. There is currently no clear consensus on which risk factors carry the most weight, or how consistently these associations apply across age groups and sexes. A deeper understanding of how each factor contributes to and is affected by OSA could help refine both screening and treatment. Clarifying the roles of sex, age, and race may lead to more personalized and effective clinical approaches.

Despite the growing body of evidence, the relationship between OSA and its associated comorbidities and risk factors remain understudied. New Orleans offers a valuable study population due to its high rates of obesity (39.9% vs 32.8% nationally), tobacco use (19.3% and 21.6% in adults aged 25–44 and 45–64 vs 12.5% and 15.1% nationally), and alcohol consumption [21–24]. This study advances prior work by evaluating a broader range of comorbidities and integrating both modifiable and non-modifiable risk factors to better understand the complex relationship between OSA and these variables. It also highlights a diverse, urban patient population in New Orleans that is underrepresented in existing OSA research.

## Methods

### Study design and participants

This study used data from electronic medical records obtained from the Otolaryngology, head and neck surgery (OHNS) department at Tulane University Medical Group (TUMG). The patients’ records were deidentified for privacy. This study was approved by the Tulane Institutional Review Board (2022–1783-TCC). Designed as a case–control study, patients with OSA, serving as cases and those without OSA (nOSA) being the control group. OSA cases were identified based on the diagnosis of OSA (ICD-10-CM code G47.33) following a home sleep study or polysomnography. We reviewed records of patients treated at the OHNS department at TUMG within the past 5 years (2019–2024; n = 19,875). Four patients were excluded from the case group because they had normal sleep study reports even though OSA was listed in their chart report. Ultimately, our case group (OSA) included 313 patients with OSA and 19,558 patients without OSA (nOSA).

Demographic information including sex, race, and age were recorded along with modifiable risk factors including body mass index (BMI), alcohol and tobacco use. Patients’ risk factors were further categorized by age (< 20, 20–39, 40–59, 60–79, 80 +), BMI (underweight, normal, overweight, obese I, obese II, obese III), tobacco use (nonsmoker, current smoker, former smoker), and alcohol use (0, 1, 2, 3, 4 points). The patient’s other comorbid conditions were recorded with the corresponding ICD codes (**Supplemental **Table 1). We recognize that coding practices may vary; to minimize misclassification errors and reduce selection bias, comorbidities were grouped into 6 categories: cardiovascular disorders (comprised of atrial fibrillation, arrhythmias other, atrial flutter, coronary artery disease, cerebrovascular disease, heart failure, hypertension, left bundle branch block, right bundle branch block), respiratory disorder (asthma, chronic obstructive pulmonary disease (COPD), pulmonary embolism (PE), pulmonary fibrosis), psychiatric disorders (depression), metabolic disorder (diabetes, dyslipidemia, gout, vitamin D. deficiency), gastrointestinal disorder (Gastroesophageal reflux disease (GERD), hepatic disease), and renal disorder (chronic kidney disease).

**Table 1:** Demographics and other characteristics of case and control patients

Characteristics	OSA (n = 313)	nOSA (n = 19,558)
Sex		
Male (%)	187 (59.74)****	9197 (47.02)****
Female (%)	126 (40.26)****	10,361 (52.98) ****
Mean Age ± SD (Range)	61.02 ± 15.40 (19–89)****	54.13 ± 19.63 (18–89)****
Under 20 (%)	1(0.32)	350 (1.80)
20–39 (%)	30 (9.58)	5081 (25.98)
40–59 (%)	104 (33.23)	5200 (26.59)
60–79 (%)	143 (45.69)	7129 (36.45)
80 + (%)	35 (11.18)	1798 (9.19)
Race		
Asian (%)	4 (1.28)	328 (1.68)
Black/AA (%)	86 (27.48)	4166 (21.30)
Hispanic (%)	1 (0.32)	51 (0.26)
White (%)	167 (53.35)	11,617 (59.40)
Other (%)	16 (5.11)	513 (2.62)
Unknown (%)	39 (12.46)	2883 (14.74)
Mean BMI ± SD	35.35 ± 8.30****	29.77 ± 44.88****
Underweight (%)	1 (0.32)	318 (1.63)
Normal weight (%)	22 (7.03)	4964 (25.38)
Overweight (%)	60 (19.17)	4960 (25.36)
Obese I (%)	78 (24.92)	3113 (15.92)
Obese II (%)	64 (20.45)	1497 (7.65)
Obese III (%)	76 (24.28)	1217 (6.22)
Unknown (%)	12 (3.83)	3489 (17.84)
Tobacco use		
Current smoker (%)	32 (10.22)	1105 (5.65)
Former smoker (%)	72 (23.00)	1035 (5.29)
Non-smoker (%)	188 (60.06)	5949 (30.42)
Unknown (%)	21 (6.71)	11,469 (58.64)
Alcohol use		
Never drinker (0, %)	1 (0.32)	26 (0.13)
Monthly or less (1, %)	87 (27.80)	2043 (10.45)
2–4 times per month (2, %)	30 (9.58)	943 (4.82)
2–3 times per week (3, %)	32 (10.22)	667 (3.41)
4 or more times per week (4, %)	16 (5.11)	464 (2.37)
Unknown (%)	147 (46.96)	15,415 (78.82)

*Abbreviations*: *AA* African American, *BMI* body mass index, *OSA* obstructive sleep apnea, *nOSA* no obstructive sleep apnea (healthy patient), *SD* standard deviation

### Statistical analysis

Statistical analyses were performed using JMP 17.2.0. To compare OSA and nOSA patients, nominal logistic regression was employed to analyze differences based on sex (male or female), race (Asian, Black/African American (AA), Hispanic, other), age group (< 20, 20–39, 40–59, 60–79, 80 +), BMI group (underweight, normal, overweight, obese I, obese II, obese III), tobacco use (nonsmoker, current smoker, former smoker), alcohol use (0, 1, 2, 3, 4 points), and comorbidities (cardiovascular, respiratory, psychiatric, metabolic, GI, renal disorders). A two-tailed t-test was used to determine differences in age, BMI, race, alcohol and tobacco use between OSA and nOSA groups. Fisher’s exact test was used to determine differences between sex using GraphPad prism 10.3.1. A significant level of 0.05 was adopted.

For each categorical comparison (sex, race, age group, BMI group, tobacco use, and alcohol use), the percentage of patients with each comorbidity of interest were calculated. For example, we totaled the number of males with cardiovascular disorders and divided by the total number of males. We then calculated a percentage by multiplying the result by 100. This was done for each category within the control and OSA case groups. These percentages were then used to generate graphs for each category of interest. Calculations and graphs were generated on Microsoft Excel.

## Results

### Patient demographic

A total of 19,871 patients were included, 313 of the patients had OSA while 19,558 did not have an OSA diagnosis. The mean (SD) age was 61.02 (15.40) and 54.13 (19.63), with a range of 19–89 and 18–89 for the OSA and nOSA groups respectively. In the OSA group 59.74% of the patients were males and 40.26% were females, while the nOSA group had 47.02% and 52.98% males and females respectively. There was a statistically significant difference in patient ages and BMI between the OSA and nOSA groups (p < 0.001) (Table 1).

### OSA prevalence and comorbidity patterns across different risk factors

After adjusting for sex, age group, BMI group, tobacco use, and alcohol use, patients with OSA were found to be more likely than controls to have the following comorbidities: cardiovascular disorders (odds ratio [OR] 23.74; 95% CI, 3.87–145.76, *p* = 0.0006), and respiratory disorders (OR 10.12; 95% CI, 3.07–33.38, *p* = 0.0001) (Table 2). Comparisons for psychiatric, metabolic, and GI disorders were not possible, as they were absent in the nOSA group.

**Table 2:** Comparative analysis of comorbidities in OSA and nOSA patients

Odds Ratio for Cardiovascular Disorders					
Level1	**Level2**	**OR**	**Prob > Chisq**	**95% CI Wald (Lower)**	**95% CI Wald (Upper)**
OSA	nOSA	23.74	0.0006*	3.87	145.76
nOSA	OSA	0.042	0.0006*	0.0069	0.26
Odds Ratio for Respiratory Disorders					
Level1	**Level2**	**OR**	**Prob > Chisq**	**95% CI Wald (Lower)**	**95% CI Wald (Upper)**
OSA	nOSA	10.12	0.0001*	3.07	33.38
nOSA	OSA	0.099	0.0001*	0.030	0.33

*Abbreviations*: *CI* confidence interval, *OSA* obstructive sleep apnea, *nOSA* no obstructive sleep apnea (healthy patient)

A higher proportion of males had OSA compared to females (OR 1.71; 95% CI, 1.05–2.79, *p* = 0.0302) (Table 3). BMI study subjects were classified as underweight (BMI < 18.5), normal weight (BMI 18.5–24.9), overweight (BMI 25–29.9), obese I (BMI 30–34.9), obese II (BMI 35–39.9), obese III (BMI ≥ 40). BMI subgroup analysis found a significantly higher OSA prevalence in all three classes of obese groups compared to the normal weight group (obese I – OR 7.07; 95% CI, 2.69–18.57, *p* < 0.0001, obese II – OR 9.65; 95% CI, 3.50–26.66, *p* < 0.0001, obese III – OR 8.81; 95% CI, 3.07–25.25, *p* < 0.0001). A higher prevalence of OSA was also observed in all three groups of obese patients relative to the overweight group (obese I – OR 3.43; 95% CI, 1.71–6.89, *p* = 0.0005, obese II – OR 4.68; 95% CI, 2.17–10.09, *p* < 0.0001, obese III – OR 4.27; 95% CI, 1.90–9.63, *p* = 0.0005) (Table 3).

**Table 3:** Comparative analysis of demographic and modifiable risk factor prevalence in OSA and nOSA patients

Sex (OR)	Male OR (95% CI; *p*-value)				Female OR (95% CI; *p*-value)		
Male	-				1.71 (1.05–2.79; 0.0302*)		
Female	0.58 (0.36–0.95; 0.0302*)				-		
**Race (OR)**	**Asian OR (95% CI; p-value)**	**Black/AA OR (95% CI; p-value)**		**Hispanic OR (95% CI; p-value)**	**Other OR (95% CI; p-value)**		**White OR (95% CI; p-value)**
Asian	-	0.87 (0.11–6.69; 0.8950)		238,745.14 (0; 0.9935)	1.13 (0.099–13.00; 0.9194)		1.06 (0.14–8.00; 0.9525)
Black/AA	1.15 (0.15–8.81; 0.8950)	-		273,875.48 (0; 0.9935)	1.30 (0.29–5.83; 0.7309)		1.22 (0.69–2.15; 0.4919)
Hispanic	0 (0; 0.9935)	0 (0; 0.9935)		-	0 (0; 0.9936)		0 (0; 0.9936)
Other	0.88 (0.08–10.11; 0.9194)	0.77 (0.17–3.44; 0.7309)		210,499.14 (0; 0.9936)	-		0.94 (0.22–4.04; 0.9310)
White	0.94 (0.12–7.08; 0.9525)	0.82 (0.47–1.44; 0.4919)		224,538.39 (0; 0.9936)	1.07 (0.25–4.60; 0.9310)		-
**Age group (OR)**	** < 20 OR (95% CI; p-value)**	**20–39 OR (95% CI; p-value)**		**40–59 OR (95% CI; p-value)**	**60–79 OR (95% CI; p-value)**		**80 + OR (95% CI; p-value)**
< 20	-	0 (0; 0.9891)		0 (0; 0.9886)	0 (0; 0.9890)		0 (0; 0.9895)
20–39	682,011.11 (0; 0.9891)	-		0.54 (0.26–1.12; 0.0969)	0.87 (0.40–1.88; 0.7237)		1.62 (0.54–4.91; 0.3918)
40–59	1,267,986.1 (0; 0.9886)	1.86 (0.89–3.87; 0.0969)		-	1.62 (0.94–2.80; 0.0843)		3.02 (1.16–7.84; 0.0235*)
60–79	783,315.7 (0; 0.9890)	1.15 (0.53–2.48; 0.7237)		0.62 (0.36–1.07; 0.0843)	-		1.86 (0.75–4.64; 0.1816)
80 +	420,444.61 (0; 0.9895)	0.62 (0.21–1.87; 0.3918)		0.33 (0.13–0.86; 0.0235*)	0.54 (0.22–1.34; 0.1816)		-
**BMI (OR)**	**Under-weight OR (95% CI; p-value)**	**Normal OR (95% CI; p-value)**	**Over-weight OR (95% CI; p-value)**	**Obese I OR (95% CI; p-value)**	**Obese II OR (95% CI; p-value)**		**Obese III OR (95% CI; p-value)**
Under-weight	-	0 (0; 0.9878)	0 (0; 0.9871;)	0 (0; 0.9859;)	0 (0; 0.9856)		0 (0; 0.9857)
Normal	190,864.79 (0; 0.9878)	-	0.48 (0.17–1.40; 0.1795)	0.14 (0.05–0.37; < 0.0001*)	0.10 (0.04–0.29; < 0.0001*)		0.11 (0.04–0.33; < 0.0001*)
Over-weight	393,573.2 (0; 0.9871)	2.06 (0.72–5.93; 0.1795)	-	0.29 (0.15–0.59; 0.0005*)	0.21 (0.10–0.46; < 0.0001*)		0.23 (0.10–0.53; 0.0005*)
Obese I	1,349,487.1 (0; 0.9859)	7.07 (2.69–18.57; < 0.0001*)	3.43 (1.71–6.89; 0.0005*)	-	0.73 (0.39–1.38; 0.3368)		0.80 (0.40–1.60; 0.5326)
Obese II	1,842,522 (0; 0.9856)	9.65 (3.50–26.66; < 0.0001*)	4.68 (2.17–10.09; < 0.0001*)	1.37 (0.72–2.58; 0.3368)	-		1.10 (0.51–2.34; 0.8136)
Obese III	1,681,749.6 (0; 0.9857)	8.81 (3.07–25.25; < 0.0001*)	4.27 (1.90–9.63; 0.0005*)	1.25 (0.62–2.49; 0.5326)	0.91 (0.43–1.95; 0.8136)		-
**Tobacco (OR)**	**Nonsmoker OR (95% CI; p-value)**			**Former smoker OR (95% CI; p-value)**	**Current smoker OR (95% CI; p-value)**		
Nonsmoker	-			2.50 (1.11–5.63; 0.0275*)	1.30 (0.62–2.71; 0.4846)		
Former smoker	0.40 (0.18–0.90; 0.0276*)			-	0.52 (0.19–1.42; 0.2037)		
Current smoker	0.77 (0.37–1.61; 0.4846)			1.92 (0.70–5.25; 0.2037)	-		
**Alcohol (OR)**	**0 point OR (95% CI; p-value)**	**1 point OR (95% CI; p-value)**		**2 points OR (95% CI; p-value)**	**3 points OR (95% CI; p-value)**	**4 points OR (95% CI; p-value)**	
0 point	-	3.49 (0.35–34.65; 0.2857)		4.40 (0.41–46.93; 0.2197)	7.26 (0.61–86.22; 0.1166)	3.53 (0.32–39.50; 0.3053)	
1 point	0.29 (0.03–2.84;0.2857)	-		1.26 (0.55–2.90; 0.5864)	2.08 (0.68–6.32; 0.1971)	1.01 (0.39–2.64; 0.9799)	
2 points	0.23 (0.02–2.42; 0.2197)	0.79 (0.34–1.83; 0.5864)		-	1.65 (0.47–5.77; 0.4342)	0.80 (0.26–2.46; 0.7009)	
3 points	0.14 (0.01–1.64; 0.1166)	0.48 (0.16–1.46; 0.1971)		0.61 (0.17–2.12; 0.4342)	-	0.49 (0.13–1.84; 0.2881)	
4 points	0.28 (0.03–3.16; 0.3053)	0.99 (0.38–2.57; 0.9799)		1.25 (0.41–3.82; 0.7009)	2.05 (0.54–7.74; 0.2881)	-	
*Abbreviations*: *AA* African American, *CI* confidence interval, *BMI* body mass index, *OSA* obstructive sleep apnea, *nOSA* no obstructive sleep apnea (healthy patient)							

Former smokers had higher OSA prevalence than nonsmokers (OR 2.50; 95% CI, 1.11–5.63, p = 0.0275). Based on age group, OSA was less prevalent in the 80 + group compared to the 40–59 age group (OR 0.33; 95% CI, 0.13–0.86, p = 0.0235). Race and alcohol use show no statistically significant difference in OSA prevalence (Table 3.).

### Characterization of comorbidities in OSA and nOSA patients by risk factors

Comorbidities were compared across OSA and nOSA groups based on sex, race, age groups, BMI subgroups, tobacco use, and alcohol consumption.

**Fig. 1:**
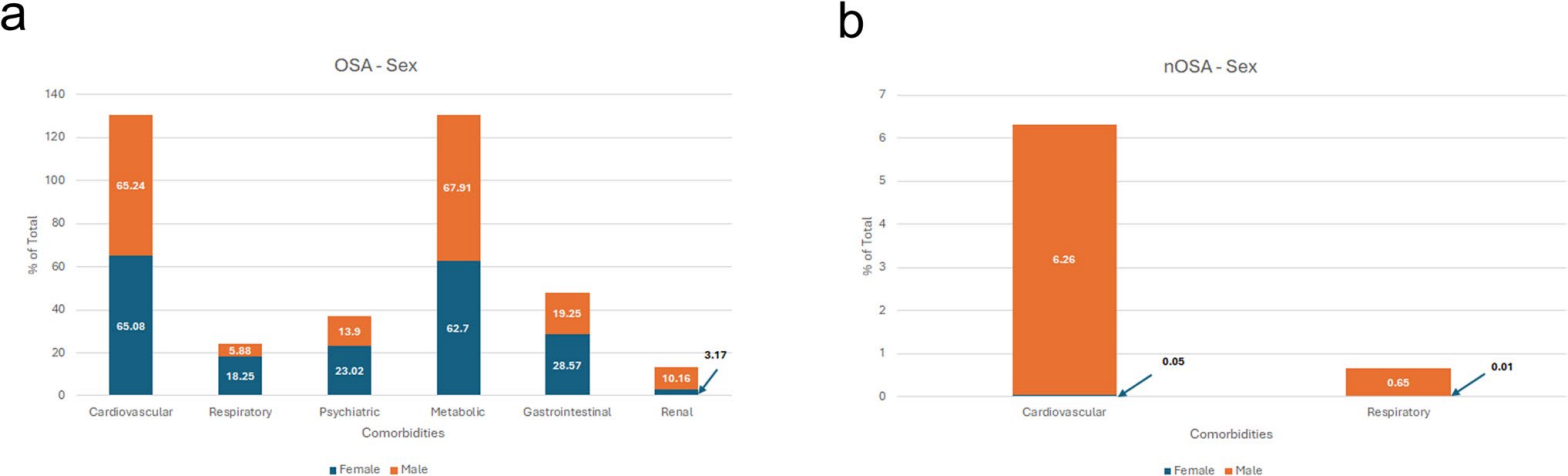
Comorbidity Prevalence in OSA and nOSA Patients by Sex. Comparison of OSA comorbidities in male and females with **a**) OSA and (**b**) without OSA

Metabolic and cardiovascular disorders were the most predominant comorbidities in both males and females with OSA. Metabolic disorders were the most common comorbidities in males compared to females (68% vs 63%), while cardiovascular disorders were equally common (65% in both sexes). Females with OSA demonstrated a higher prevalence of GI disorders (29% vs 19% in males), psychiatric disorders (23% vs 14% in males), and respiratory disorders (18% vs 6% in males). Males with OSA had a higher prevalence of renal disorder (10% vs 3%) (Fig. 1a). In the control group, males exhibited a higher burden of comorbidities, with cardiovascular disorders being more prevalent than respiratory diseases (6.26% vs 0.65%). Although females in the control group followed a similar trend (0.05% vs 0.01%), they had lower overall comorbidity rates (Fig. 1b).

**Fig. 2:**
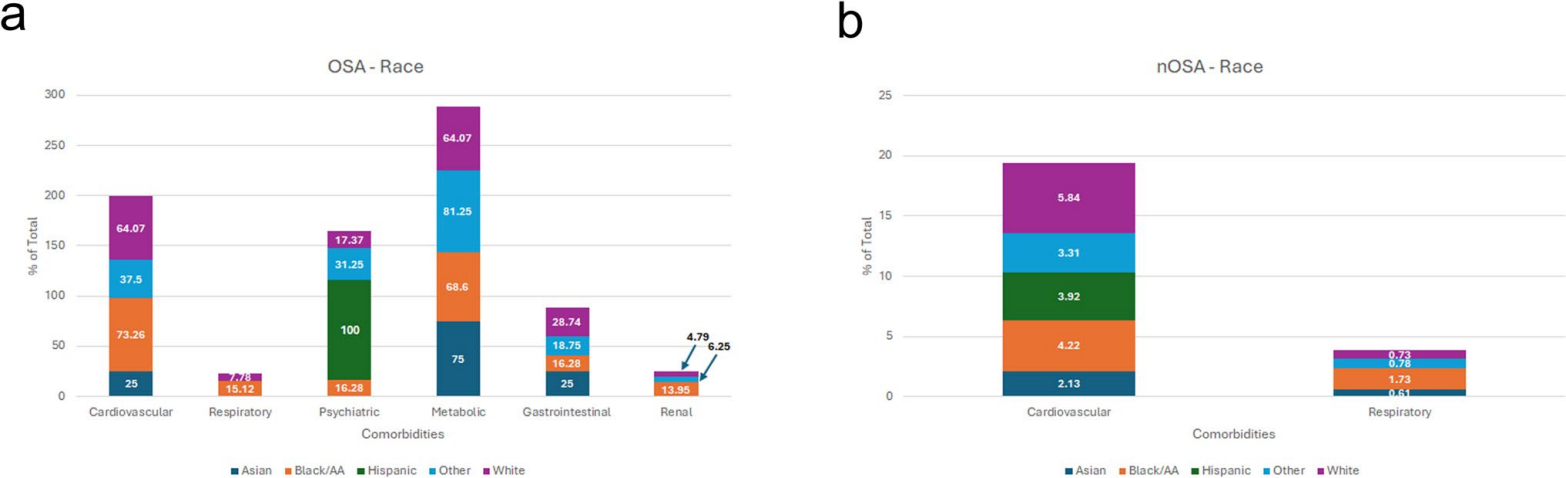
Comorbidity Prevalence in OSA and nOSA Patients by Race. Comparison of OSA comorbidities among different races with **a**) OSA and (**b**) without OSA

Comorbidity prevalence varied among racial groups with OSA. Among Asians with OSA, metabolic disorders were the most prevalent comorbidity (75%), followed by cardiovascular (25%) and GI (25%) disorders. In Black or African American (AA) individuals with OSA, cardiovascular disorders (73%) were the most prevalent, followed closely by metabolic disorders (69%). In White individuals with OSA, there was an equal prevalence of metabolic and cardiovascular disorders (64%). The Hispanic individual with OSA (n = 1) had psychiatric disorder as the only comorbidity. Individuals classified as “Other” with OSA showed metabolic disorders as the most prevalent comorbidity (81%), followed by cardiovascular disorders (38%) (Fig. 2a). In the nOSA control groups, cardiovascular disorders were more prevalent than respiratory disorders across all races (Fig. 2b).

**Fig. 3:**
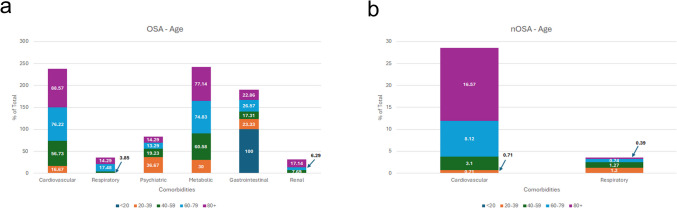
Comorbidity Prevalence in OSA and nOSA Patients by Age. Comparison of OSA comorbidities in different age groups with **a**) OSA and (**b**) without OSA

Next, we explored comorbidities across different age groups. In patients with OSA under 20 years old (n = 1), GI disorders were the most common. In the 20–39 age group with OSA, psychiatric disorders were the most prevalent. Older individuals (age 40 and above) experienced higher rates of cardiovascular and metabolic disorders compared to younger patients. These comorbidities increased progressively with age groups > 40 years (Fig. 3a). A similar trend was observed in the control population, where cardiovascular disorders were the most prevalent in older patients (age 40 and above), with the prevalence doubling from one age group to the next. Respiratory disorders were more prevalent among the control population aged 20–39 (1.20% vs 0.71% for cardiovascular disorders) (Fig. 3b).

**Fig. 4:**
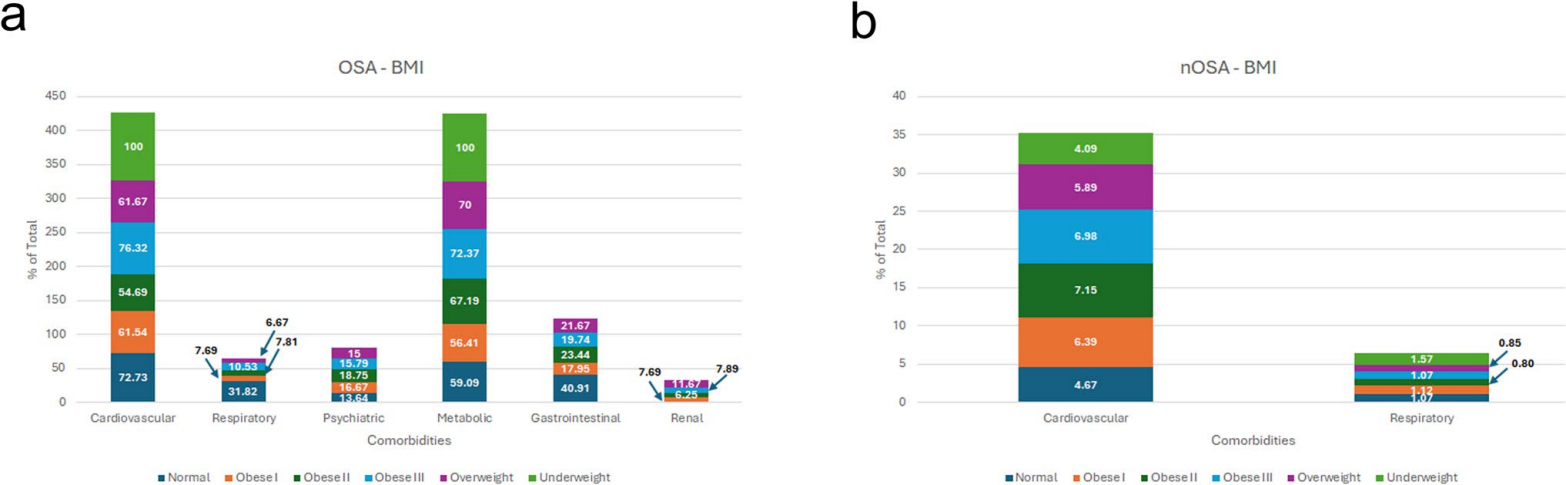
Comorbidity Prevalence in OSA and nOSA Patients by BMI. Comparison of OSA comorbidities among different BMI categories with **a**) OSA and (**b**) without OSA

When we compared different BMI subgroups, the underweight patient with OSA (*n* = 1) had cardiovascular and metabolic disorders as the most prevalent comorbidities (100%). Patients with normal weight with OSA had higher rates of cardiovascular disorders (73%), followed by metabolic disorders (59%). Overweight patients with OSA presented with metabolic disorders (70%), followed by cardiovascular disorders (62%). While cardiovascular disorders were the most prevalent in obese I (62%) and obese III (76%) patients, metabolic disorders were the most prevalent in obese II patients (67%) (Fig. 4a). In the control group, cardiovascular disorders were the most prevalent, showing an almost linear relationship between increased BMI and the percentage of cardiovascular disorders. This increase did not continue beyond obese II, as there was no difference between obese II and obese III patients (Fig. 4b).

**Fig. 5:**
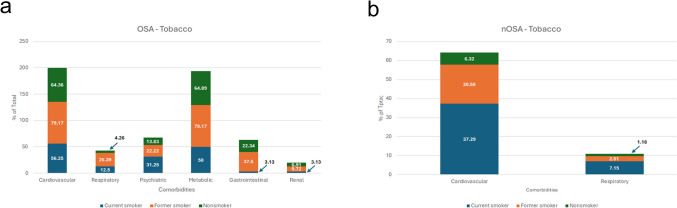
Comorbidity Prevalence in OSA and nOSA Patients by Tobacco use. Comparison of OSA comorbidities with **a**) OSA and (**b**) without OSA

Cardiovascular and metabolic disorders are the most prevalent comorbidities when comparing tobacco use in the OSA population. Former smokers exhibited a higher prevalence of cardiovascular disorder (79%) compared to current smokers (56%) and non-smokers (64%). Former smokers also showed a higher prevalence of metabolic disorders (79%) compared to current smokers (50%), and non-smokers (65%). Current smokers had lower prevalence of most diseases compared to non-smokers and former smokers, with the exception of psychiatric disorders (Fig. 5a). In the control group, cardiovascular disorders are more prevalent, with current smokers (37%) having the highest prevalence, compared to former smokers (21%), and non-smokers (6%) (Fig. 5b).

**Fig. 6:**
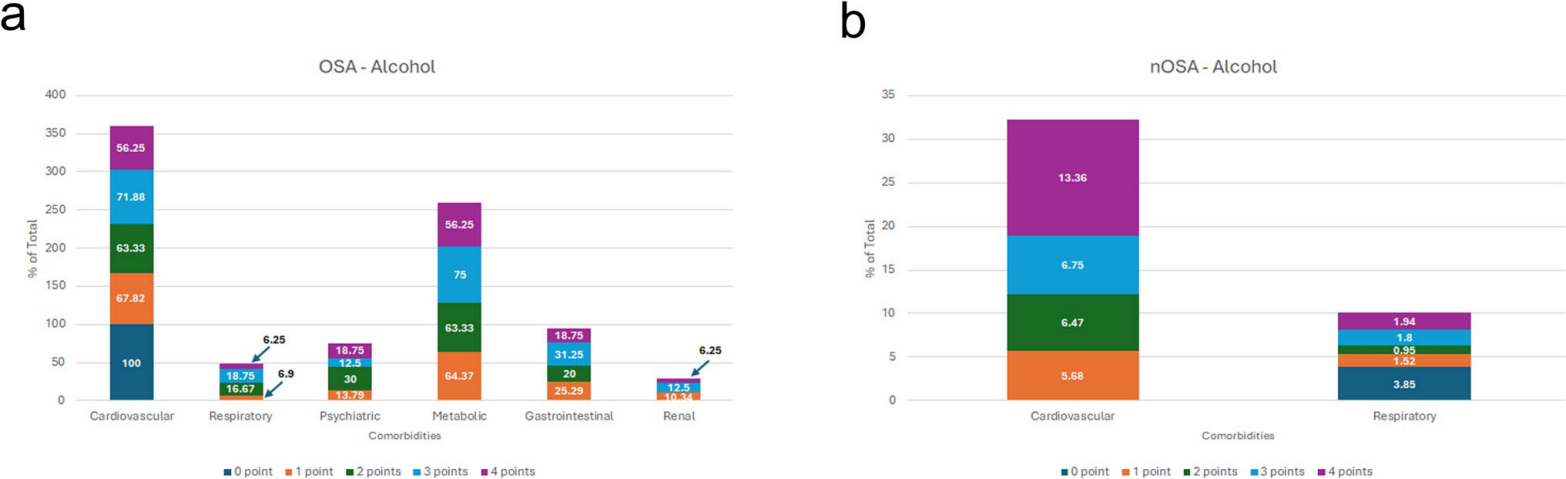
Comorbidity Prevalence in OSA and nOSA Patients by Alcohol use. Comparison of OSA comorbidities with **a**) OSA and (**b**) without OSA

Finally, when we examined the relationship between comorbidities and alcohol usage in patients with OSA, cardiovascular and metabolic disorders were the most prevalent across the group. Cardiovascular disorder was most prevalent in non-drinkers (0-point; *n* = 1, 100%) compared to patients who drink monthly or less (1 point; 68%), 2–4 times per month (2 points; 63%), 2–3 times per week (3 points; 72%), and 4 or more times per week (4 points; 56%). Metabolic disorder was most common in the 3 points group (75%), compared to 1 point (64%), 2 points (63%), and 4 points (56%) (Fig. 6a). In the nOSA control group, cardiovascular disorders were the most prevalent, with the prevalence increasing with higher alcohol consumption (Fig. 6b).

## Discussion

To our knowledge, this study is the first to examine OSA comorbidities alongside demographic and modifiable risk factors in the New Orleans population. Our findings reinforce existing evidence linking cardiovascular [7, 12–15] and respiratory [7, 16, 17] disorders to OSA while also expanding the list of associated comorbidities to include renal, gastrointestinal, metabolic, and psychiatric disorders, consistent with prior studies[9]. While the connection between metabolic and cardiovascular diseases and OSA is well established [13, 18, 25], our results suggest that a broader spectrum of comorbidities may contribute to the pathogenesis of OSA. Although the exact causal relationship between OSA and these disorders remain unclear, the increased OR for cardiovascular and respiratory disorders, along with the presence of other comorbidities exclusively in OSA patients, indicate OSA may serve as an independent risk factor.

Our analysis emphasized that demographic and modifiable risk factors significantly influence OSA risk. Males had twice the odds of developing OSA compared to females, consistent with prior studies [2, 6, 9]. Sex differences in OSA manifestations suggest males may experience more severe outcomes [26]. While our study did not track disease outcomes by sex, the higher OSA risk in males may be due to greater visceral fat mass [27], contributing to upper airway collapsibility, ventilatory control instability, and higher arousal thresholds. Underdiagnosis in women with atypical symptoms or protective physiological factors may also play a role. Future research should explore sex-specific screening and treatment approaches for OSA.

Age is a known risk factor for OSA, with prevalence increasing independently of other risk factors [2, 28], However, some reports suggest that the frequency of OSA declines with age [28]. Our results show a statistically significant difference in OR between individuals aged 40–59 and those aged 80 +, with OSA decreasing after 59, though not statistically significant (Table 3.). This suggests a higher disease burden in middle-aged individuals, warranting further investigation. Since BMI peaks at 70–79 before declining [27], given the association between OSA and obesity [25], the observed drop in OSA prevalence in older adults may be linked to BMI reduction. Further analysis of age-associated BMI changes and OSA severity could clarify this association. Ramezani et al. [29] report that while the prevalence of severe sleep apnea increases with age, all-cause mortality among these patients decreases, particularly in those over 65 years. Similarly, Marti et al. found that severe sleep apnea is associated with excess mortality primarily in individuals under 50 [30]. These findings may reflect survivorship bias, as patients with severe OSA may die earlier in life, and competing mortality risks in older adults, where other comorbidities contribute more significantly to mortality. This may help explain the lower observed frequency of OSA with age in our study. The relationship between OSA and age remains unclear, as some studies suggest that the clinical impact of OSA in older-onset patients (> 65 years), even with similar AHI levels to those with middle-age onset, may be milder [31], highlighting the need for further investigation.

OSA has classically been linked to obesity [26, 32, 33], a condition marked by dysregulated energy metabolism and adipose tissue inflammation [34]. OSA can exacerbate the metabolic disturbances seen in obesity by influencing insulin resistance, glucose metabolism, and gut microbiota [9]. Our findings support this connection, showing that a high BMI (≥ 30) increases the likelihood of developing OSA. However, beyond the threshold of obesity, further increases in BMI did not significantly raise the risk of OSA. This suggests that the relationship between obesity and OSA is not linear, underscoring the complex interaction between the two conditions.

Cigarette smoking is a modifiable risk factor for cardiovascular disease, though its association with OSA remains inconclusive [3, 35, 36]. Our findings show former smokers have significantly higher odds of OSA than nonsmokers and current smokers. Prior meta-analysis suggest longer smoking duration and heavy smoking correlate with severe OSA [3]. Since our study did not account for smoking duration, former smokers may have had a longer, heavier smoking history, explaining the observed results. Future studies examining smoking duration and severity could clarify this relationship. Nevertheless, our findings support an overall association between smoking and OSA (Table 3).

Chiang et al. found that sleep apnea patients with any comorbidity had a higher mortality risk, with hypertension, chronic obstructive pulmonary disease (COPD), and diabetes significantly increasing this risk [7]. Our study examined comorbidity distribution across demographics and modifiable risk factors, revealing broader and more diverse health issues in OSA patients. Cardiovascular and metabolic disorders were most prevalent, with cardiovascular conditions substantially more common in OSA than in the general population (Fig. 1–6), aligning with prior studies [12–14, 25, 26, 33]. Other comorbidities varied by sex, race, BMI, tobacco use, and alcohol use. Notably, females with OSA exhibited a higher prevalence of GI, psychiatric, and respiratory disorders, whereas males had more metabolic and renal disorders (Fig. 1). These sex-specific differences, absent in controls, suggest the potential sex-specific mechanisms in OSA. OSA has been reported with increased prevalence in patients with psychiatric conditions such as post-traumatic stress disorder (PTSD), major depressive disorder, bipolar disorder and schizophrenia [37–39]. In females with chronic PTSD, OSA and other sleep-disordered breathing may present as insomnia and be misdiagnosed [38]. Although this study did not assess age-related differences in OSA severity among females, the observed sex-specific differences may be influenced by hormonal factors and menstrual cycle status, as OSA is more common in postmenopausal women and those with irregular menstrual cycles [40]. Given atypical presentation of OSA in females, understanding sex-specific differences may improve recognition and diagnosis in this population.

Comorbidity prevalence varied by race, age, and lifestyle factors. Black/AA individuals had the highest rates of renal, respiratory, and cardiovascular conditions, aligning with previous findings of increased OSA severity and risk factors in this population [41]. While race was not an independent risk factor for OSA, socioeconomic factors may contribute to comorbidity distribution.

Younger OSA patients predominantly had GI disorders, while cardiovascular and psychiatric disorders were more prominent in older age groups, highlighting the need for targeted interventions and age-specific screening.

Modifiable risk factors, particularly obesity and smoking, contributed to the overall comorbidity burden in the OSA population. When evaluating modifiable risk factors, metabolic and cardiovascular disorders remain highly prevalent, with GI, respiratory, and psychiatric disorders also showing a substantial presence. This underscores the importance of addressing these comorbidities in the context of OSA, as they have been similarly associated with OSA in other studies [9].

While our findings highlight critical associations, several limitations must be considered. Although we tried to limit selection bias resulting from ICD-10 code misclassification, the variability in coding practices could have introduced bias in this study. The lack of sleep study records for all patients prevented assessment of OSA severity. Differences in age, sex, and BMI between the OSA and control groups may have influenced results, and missing data on modifiable risk factors reduced statistical power. The smaller OSA sample size may have limited the detection of certain associations. Furthermore, the study’s focus on a single health system in New Orleans also limits its generalizability to a broader population. Incomplete records on CPAP usage and adherence also made it difficult to evaluate the impact of treatment on comorbidities.

Despite these limitations, our findings highlight the increased risk of cardiovascular and respiratory disorders in OSA patients compared to nOSA patients. Males, middle-aged individuals (aged 40–59 years), individuals with a BMI > 30, and former smokers are at higher risk for OSA. Cardiovascular and metabolic disorders are the most prevalent disease comorbidities, but the presence of other comorbidities such as GI, psychiatric, respiratory, and renal disorders emphasize the need for comprehensive care in OSA patients. Healthcare providers should prioritize monitoring both well-established and under-recognized comorbidities to help mitigate the extensive morbidity associated with this condition.

## Conclusion

In conclusion, our study underscores the complex relationship between OSA and a broad spectrum of comorbidities, influenced by demographic and modifiable risk factors. By identifying increased risks for OSA and associated conditions, our findings emphasize the need for comprehensive healthcare strategies targeting both well-established and under-recognized comorbidities to optimize patient outcomes.

Our study also highlights several areas for future research. To improve clinical management of OSA, future studies should examine whether the modifiable and non-modifiable risk factors identified here are causally linked to the development and progression of OSA. It is also important to investigate whether consistent OSA management alters the trajectory of associated comorbidities over time. Additionally, exploring age-specific and sex-related differences in OSA comorbidities may offer valuable insights for tailoring treatment across diverse populations.

## Disclosures

We employed AI tools, including Grammarly for Microsoft Office, ChatGPT, and Gemini, to assist in revising sections of this article, with the sole aim of improving clarity and readability. However, all AI-generated content underwent thorough review, editing, and final verification by the authors.

## Supplementary Information

Below is the link to the electronic supplementary material.

Supplementary file 1: Supplemental Table 1. ICD-Codes of Disease and Comorbid Conditions.

## Data Availability

The participants of this study did not give written consent for their data to be shared publicly, so due to the sensitive nature of the research supporting data is not available.
