# Supplementary material for: Demographic and modifiable risk factors impacting obstructive sleep apnea comorbidities: a New Orleans case–control study
Source: Sleep Breath. 2025 Jun 6;29(3):204. doi: 10.1007/s11325-025-03379-z (PMC12143987; doi:10.1007/s11325-025-03379-z)
Supplement: Supplementary file 1 — Supplemental Table 1. ICD-Codes of Disease and Comorbid Conditions. [file 11325_2025_3379_MOESM1_ESM.docx]

| **Disease and Comorbidities** | **ICD Codes** |
| --- | --- |
| OSA (Patient has OSA) | **G47.33** |
| Atrial Flutter | **I48.3, I48.4, I48.92** |
| Atrial Fibrillation | **I48.0, I48.2, I48.11, I48.19, I48.20, I48.91** |
| Arrhythmias other | **I47.1, I47.2, I49.5** |
| Asthma | **J45.909** |
| Coronary Artery Disease | **I25.2,** **I25.5, I25.10, I25.110, I25.119, I25.810, 413.9** |
| Cerebrovascular Disease | **I63.9, I63.50, I63.512, I67.89, G45.9, Z86.73**, |
| Chronic Kidney Disease | **N18.2, N18.3, N18.4, N18.6, N18.9, N18.31** |
| COPD | **J44.9** |
| Depression | **F32.A, F32.2, F32.9, F32.89** |
| Diabetes | **E11.9, E11.29, E11.40, E11.59, E11.69, Z86.39** |
| Dyslipidemia | **E78.00, E78.2, E78.5, E78.89** |
| GERD | **K21.9** |
| Gout | **M10.9, Z87.39** |
| Hepatic disease | **K76.0** |
| Heart Failure | **I50.9, I50.20, I50.30** |
| Hypertension | **I10** |
| Ischemic Heart Disease | **I25.5** |
| LBBB | **I44.7** |
| Pulmonary Embolism | **I26.99** |
| Pulmonary Fibrosis | **J84.10** |
| Pulmonary Hypertension | **I27.2** |
| RBBB | **I45.10** |
| Vit. D Deficiency | **E55.9** |
